# Diagnosis of extraintestinal pathogenic *Escherichia coli* pathogenesis in urinary tract infection

**DOI:** 10.1016/j.crmicr.2024.100296

**Published:** 2024-10-21

**Authors:** Deenadayalan Karaiyagowder Govindarajan, Biniam Moges Eskeziyaw, Kumaravel Kandaswamy, Degisew Yinur Mengistu

**Affiliations:** a Research Center for Excellence in Microscopy, Department of Biotechnology, Kumaraguru College of Technology, India; b Department of Biotechnology, Debre Berhan University, Debre Berhan, Ethiopia; c Institute of Biotechnology, University of Gondar, Gondar, Ethiopia

**Keywords:** ExPEC, UTI, Surface proteins, Diagnosis

## Abstract

Extra-intestinal pathogenic *Escherichia coli* (ExPEC) is a virulent pathogen found in humans that causes the majority of urinary tract infections, and other infections such as meningitis and sepsis. ExPEC can enter the urinary tract through two modes: ascending from the bladder or descending from the kidneys. Human anatomical structures generally prevent the transmission of pathogens between the extra-intestinal area, kidneys, bladder, and urinary tract. However, adhesins, a virulence protein of ExPEC, promote the initial bacterial attachment and invasion of host cells. In addition to adhesion proteins, ExPEC contains iron acquisition systems and toxins to evade the host immune system, acquire essential nutrients, and gain antibiotic resistance. The presence of antibiotic-resistant genes makes treating ExPEC in urinary tract infections (UTIs) more complicated. Therefore, screening for the presence of ExPEC among other uropathogens in UTI patients is essential, as it can potentially aid in the effective treatment and mitigation of ExPEC pathogens. Several diagnostic techniques are available for detecting ExPEC, including urine culture, polymerase chain reaction, serological testing, loop-mediated isothermal amplification, and biochemical tests. This review addresses strain-specific diagnostic techniques for screening ExPEC in UTI patients.

## Highlights

• ExPEC does not cause infection in the intestine which escapes from the intestine and causes infection in Urinary Tract.
• ExPEC strains were viable in the intestine without any symptoms.
• ExPEC infections in the urinary tract can be diagnosed by culture method, molecular detection, loop-mediated isothermal amplification, and immunological assay.

## Graphical abstract

**Figure:**
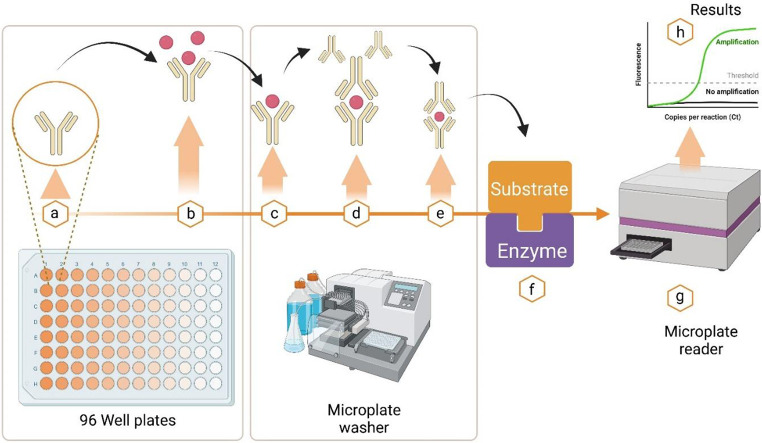

## 1 Introduction

*E. coli* is genetically diverse, comprising a wide range of serotypes that include both beneficial and harmful strains. The harmful strains can cause both intestinal and extra-intestinal infections in humans and animals. For example, the serotype O157:H7, known as Entero-Haemorrhagic *E. coli* (EHEC), is responsible for many intestinal infections which are not diagnosed in extra-intestinal infections, i.e., the infections were caused by Extra-intestinal *E. coli* (ExPEC) (Mengistu and Mengesha, 2023; Frank et al., 2011). Among *E. coli* serotypes, such as Enteropathogenic *E. coli* (EPEC), Enterohemorrhagic *E. coli* (EHEC), Enterotoxigenic *E. coli* (ETEC), Entero-invasive *E. coli* (EIEC, including Shigella), Enteroaggregative *E. coli* (EAEC), and Diarrhoeagenic Adherent *E. coli* (DAEC) (Govindarajan et al., 2020), ExPEC infections attracted global attention due to its multidrug resistance. ExPEC is further subcategorized into sepsis-causing *E. coli* (SEPEC), neonatal meningitis *E. coli* (NMEC), and uropathogenic *E. coli* (UPEC). ExPEC initiates pathogenesis after escaping from the intestine and gut commensal reservoir by utilizing virulence factors such as *pap, sfa, foc, dr, afa, kps*, and *iut* genes (Russo and Johnson, 2000). *E. coli* is responsible for approximately 90 % of urinary tract infections (UTIs) and is also associated with catheter-associated urinary tract infections (CAUTI) (Brede and Shoskes, 2011; Foxman and Brown, 2003). In the host urinary tract, ExPEC establishes its niche through toxin-antitoxin (TA) systems in prokaryotes which is extensively studied in the mouse model. These systems comprise a cognate unstable antitoxin and a stable toxin, which are ubiquitous in bacteria genomes such as *maz* and *rel* (Gerdes, Christensen, and Løbner-Olesen, 2005). These TA systems promote most of the bacterial functions such as biofilm formation, cell division, and phage resistance (Norton and Mulvey, 2012). Another *in-vitro* study showed that iron acquisition systems in the *E. coli* strain promote bacterial colonization and biofilm formation through the acquisition of extracellular iron through siderophore, (Govindarajan et al., 2022; Keogh et al., 2018; Shanmugasundarasamy et al., 2022). Similarly, studies have shown that iron supplementation facilitates bacterial colonization and intensified inflammatory responses by raising C-reactive protein levels and increasing the expression of TNF-α and IL-1β in response to *E. coli* O157:H7 (Yu et al., 2019). Asymptomatic colonization is also the hallmark of ExPEC pathogens, resulting in urinary tract infections During asymptomatic colonization, numerous ExPECs present in urinary bladder casing disease without any symptoms (Stork et al., 2018). Carriage of multiple transition metal acquisition systems UPEC suggests that the human urinary tract manipulates metal-ion availability in many ways to resist infection ExPEC also exhibits resistance to common antibiotics like trimethoprim-sulfamethoxazole, fluoroquinolones, and cephalosporins (Foxman, 2010; Govindarajan and Kandaswamy, 2023; Kadirvelu et al., 2024; Sivaramalingam et al., 2024).

Gaps in antimicrobial studies made researchers findings on ExPEC, developing methodologies and new technologies such as multi-locus enzyme electrophoresis, polymerase chain reaction (PCR), multi-locus sequence typing, pulse-field gel electrophoresis, and whole genome sequencing to understand ExPEC lineages. In 1984, Ochman and Selander pioneered a collection of 72 different *E. coli* reference strain collections (ECOR) (Ochman and Selander, 1984). Multilocus sequence typing subsequently grouped ExPEC into distinct categories among other *E. coli* serotypes (Clermont et al., 2000).

Further studies revealed that ExPEC strains possess specific antibiotic-resistant genes, complicating treatment and posing significant public health challenges (François et al., 2016). Previous reviews have covered *E. coli* pathogenesis in the intestine (Govindarajan et al., 2020), its pilus assembly mechanism (Shanmugasundarasamy et al., 2022; Ragupathi et al., 2024), and host-uropathogenic *E. coli* interactions (Govindarajan and Kandaswamy, 2022). This article focuses on the migration of ExPEC from the intestine to the urinary tract and kidneys, its antibiotic resistance, virulence factors, host-pathogen interactions, colonization, biofilm formation, adhesins, iron regulation, cell invasion, intracellular invasion, and host immune evasion in humans.

Various diagnostic methods, such as bacterial cultures, immunodiagnostic tests, and molecular techniques, have been used (Dobrut et al., 2022). However, the establishment of rapid, specific, sensitive, and cost-effective diagnostic tests for detecting ExPEC, especially in UTI cases, remains insufficient. This review discusses the current state of knowledge, provides an overview of diversity, and offers recommendations for laboratory diagnosis of ExPEC infections in terms of management, cost, labor, and feasibility. Identifying unique virulence genes or markers may provide new insights into developing or optimizing state-of-the-art molecular diagnostics that can be used at the point of care in routine clinical healthcare systems.

## 2 Pathogenesis of extra-intestinal pathogenic *E. coli*

**Fig. 1:**
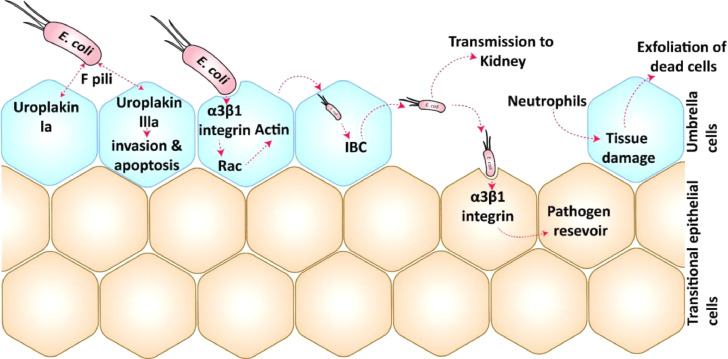
**Mechanism of Host-Pathogen Interaction:** The UPEC bacterial cells initiate their interaction with host uroepithelial cells through F pili, leading to the formation of an IBC, which causes cell apoptosis and invasion. The F pili also interact with α3β1 integrin receptors in the uroepithelial cells, disrupting actin filament arrangements and the host immune system. This alteration of actin fibers allows the bacterial cells to enter the host uroepithelial cells easily and form an IBC. The IBC then releases planktonic bacterial cells to transitional epithelial cells and kidneys. These UPEC cells establish a pathogen reservoir in the transitional epithelial cells, causing recurring UTIs in the host.

*E. coli* pathotypes possess several virulence strategies to invade a healthy host. These pathogens use fimbriae or pili and surface proteins to initiate adhesion to host cells. Following this host-pathogen interaction, *E. coli* cells disrupt host signaling pathways and evade the host immune response through predominant bacterial colonization, leading to infection (Govindarajan and Kandaswamy, 2022). Each *E. coli* pathotype has its unique pathogenic mechanism in humans, with the specific ExPEC-UPEC pathogenicity illustrated in Fig. 1.

ExPEC strains escape from the intestine and reach the urinary tract to initiate infection. These pathogens exploit the nutrient availability, such as amino acids and carbon sources, in the urinary tract to establish infection (Mann et al., 2017). Upon emerging in the urinary tract, UPEC begins its bacterial attachment using FimH pili proteins found on the tip of the F pili or Type 1 pili. The FimH proteins interact with glycosylated uroplakin-1a on the surface of the urinary bladder. Recent findings indicate that FimH uroplakin-3a interactions trigger phosphorylation signaling pathways in host cells, leading to cell invasion and apoptosis (Shanmugasundarasamy et al., 2022).

Also, the FimH interaction with beta1(β1) and alpha3 (α3) integrins disintegrates the stability and rearrangement of the actin by GTPase and kinases resulting in the formation of the Intracellular Bacterial Community (IBC) or biofilms (Eto et al., 2007), are the long filaments that evade engulfment by neutrophils and the bacteria flux out of epithelial cells. β1 and α3 integrins induce a cascade of signaling that results in internalization of the bacteria (Justice et al., 2006). These biofilms periodically release motile planktonic bacterial cells that infect surrounding cells and bladder cells, initiating an innate immune response. This immune response results in the secretion of neutrophils, causing apoptosis and exfoliation of dead cells from the bladder (Fig. 1) (Eto et al., 2007).

### 2.1 Virulence factors of ExPEC and its role in host-pathogen interaction

The virulence factors of ExPEC are classified into adhesins, invasions, iron uptake systems, protection, and toxins. The virulence factors and its genes in UPEC such as F pili (*fim*), afimbriae (*afa*), Dr fimbriae (*dra*), P-pili (*pap*), S fimbriae (*sfa*), F1C fimbriae (*foc*), curli fiber (*crl, csg*), aerobactin (*iuc, aer*), salmochelin (*iroN*), ChuA, Hma (*chu, hma*), SitABC (*sitA, B, C*), Outer membrane protein (*ompA*), serin protease autotransporter (*pic*), secreted autotransporter toxin (*sat*), vacuolating autotransporter toxin (*vat*), hemolysin A (*hlyA*), and cytotoxic necrotizing factor (*cnf*) (Sarowska et al., 2019). Apart from fimbriae and pili, other virulence surface proteins also facilitate bacterial adhesins, biofilm formation, invasions, and other pathogenesis mechanisms (Sun et al., 2022).

### 2.2 Adherence surface proteins

Type 1 pili also known as P pili are encoded with *fim* (*fimABCDEFGHIS*), the pilus assembly mechanism for the *E. coli* strains was extensively reviewed in a recent article. Each *fim* gene encodes its pilin proteins with distinct pili mechanisms (Govindarajan and Kandaswamy, 2022). For instance, the FimH was the tip pilin located at the end of F pili which binds to the mannose-binding receptors in the host cell surface and promotes the internal bacterial community (Rahdar et al., 2015). Similarly, another pili, type 2 pili (P pili) encoded by *pap* (*papABCDEFG*) in which papG was located at the tip of the P pili. The papG was differentiated into papGI, papGII, and papGIII and binds to globotriaosylceramide in epithelial cells, globose, and isoreceptors, respectively in the urinary tract (Spencer et al., 2015). Interestingly, studies have demonstrated and revealed that *pap* and *fim* genes abandon coexpression. For instance, *pap* expression abandons the expression of the *fim* gene and vice-versa (Govindarajan and Kandaswamy, 2022).

The afimbrial and Dr fimbriae are the superficial virulence proteins known as Afa/Dr fimbriae. Interestingly, the Afa fimbriae encoded by *afaABCDE* genes were similar to Dr adhesion genes such as *draABCDE*, both genes hold the same functions such as protein expression, chaperone-usher pathway, and the tip fimbriae. The Afa/Dr fimbrial tip proteins interact with the decay-accelerating factors produced in the erythrocytes and epithelial cells in the urinary tract (Servin, 2014).

Curli was the first identified as fimbria which are amyloid extracellular protein fibres present in *E. coli*. It promotes bacterial colonization, host-pathogen interaction, immune evasion in the host, and biofilm formation. The curli-mediated rugose biofilms (through localization in the air-colony interface regions) aid oxidative resistance only to biofilms rather than planktonic cells. The curli fibers were encoded with *csgABCDEFG*, synthesis of the curli proteins on the extracellular bacterial surfaces. The host-pathogen interaction of the curli fibers was to be studied (Luna-Pineda et al., 2019).

The prevalence of S fimbriae was found in 50 % ExPEC in UTI and 50 % other *E. coli* strains such as NMEC (24 %), APEC (Avian Pathogenic *E. coli*) (10 %), and DAEC (16 %) (Johnson et al., 2001). These S fimbria in the ExPEC specifically bind to human epithelium cells such as vascular endothelium cells in kidney tissues, intestinal capillary endothelium cells, and visceral epithelium cells (Korhonen et al., 1986). Specifically, S fimbria of *E. coli* binds to sialic acid or sialyl galactosides (neuraminic acid) receptors present in host epithelium cells (Parkkinen et al., 1983). This S fimbria is morphologically similar to P pili, the S fimbria is composed of sfa protein and *sfa* genetic elements (*sfaA, BCGHS*). These *sfa* genes were homogeneous genetic structures involved in several fimbrial functions such as the synthesis of fimbriae subunits, production of fimbriae, transcription, and adhesion to host cells (Hacker et al., 1993). The sfa proteins sfaA are the major fimbrial subunit encoded by *sfaA* genes, these *sfaA* genes are regulated based on environmental conditions such as osmolarity, temperature, and availability of glucose on host cells (Hacker et al., 1993). Secondly, the fimbrial formation is regulated by *sfaB* and *sfaC* genes which produce sfaB and sfaC stalk proteins (Balsalobre et al., 2003). In addition, minor fimbrial subunits such as sfaG and sfaH subunits were present along the length of S fimbriae. Finally, the sfaS fimbrial protein present in the fimbrial tip interacts with sialic acid receptors in the host for bacterial adhesion (Table 1) (Morschhäuser et al., 1990).

**Table 1:** Functions of surface proteins and virulence proteins in *E. coli.*

Pili/Surface proteins	Virulence proteins	Functions	Reference
F pili	Fim	Bacterial colonization on the urinary tract and biofilm formation.	(Rahdar et al., 2015)
P pili	pap	Promotes cytokine production by T lymphocytes and bacterial colonization.	(Spencer et al., 2015)
Afimbrial adhesin	afa	Binds to host cell receptors such as CAECAM and DAF	(Servin, 2014)
Dr fimbriae	dra		
S fimbriae	Sfa	Promotes bacterial interaction and penetration into the kidney and urinary tract cells	(Govindarajan and Kandaswamy, 2022)
Curli fiber	Crl, csg	Promotes biofilm formation and pathogenicity.	(Luna-Pineda et al., 2019)
Iha / siderophore	iha, iroN, chu, hma, and sit	Uptakes the extracellular iron and promotes bacterial colonization, biofilm formation, and pathogenicity.	(Mike et al., 2016)
F1C fimbriae	foc	Binds to renal epithelial cells (bladder) and endothelial cells (kidney).	(Subashchandrabose and Mobley, 2017)
Outer membrane protein	Omp	Promotes intracellular survival in host and immune evasion.	(Dehghani et al., 2016)
Serin protease	Pic	Damage the host epithelial cell membrane and facilitates bacterial colonization.	(Welch, 2017)
Secreted autotransporter toxins	sat	Cytotoxicity creates pores on the host cell membrane.	(Welch, 2017)
Hemolysin A	hlyA	Create pores on the host cell membrane.	(Nhu et al., 2019)
Vaculating autotransporter protein	vat	Create vacuolization on host cells	(Nichols et al., 2016)
Cytotoxic necrotizing proteins	cnf	Cell necrosis.	(Welch, 2017)

### 2.3 Outer membrane proteins

ExPEC cell membrane lipoproteins and outer membrane proteins (OmpA) hold several functions such as membrane stability, host-pathogen interaction, and active/passive transportation of molecules into/out of cells which promote the immune defense mechanism (Khalid et al., 2008). Interestingly, *pap* co-express with *omp* genes, i.e., deletion of *ompA* genes suppresses the expression of P pili which also reduces host-pathogen interaction (Teng et al., 2006). Previous studies on the OmpA protein expression studies have shown bacterial interaction with the epithelial cells on the mucosal surfaces (Krishnan and Prasadarao, 2012; Meganathan et al., 2023). Once after entering the host cell, the bacterial cells produce C4B regulatory binding proteins which possess complement binding to host serum proteins and antibodies (Abreu and Barbosa, 2017). In addition, OmpA reduces the NF-kB activation and results in immune evasion in the host. A comparison between wild-type *E. coli* and ompA-deficient *E. coli* mutants showed that wild-type *E. coli* possessed increased adherence to urinary tract and bladder epithelium cells than mutant strains. Researchers demonstrated that wild-type *E. coli* is more resistant to antibacterial peptides such as lactoferricin and collectins, whereas *ompA* deficient *E. coli* mutants show a permeable cell membrane and result in bacterial cell death (Erman et al., 2012). From the aforementioned studies, it is clear that OmpA membrane proteins possess dual roles such as preventing ExPEC from antimicrobials and delivering toxin peptides to suppress the host immune mechanism. So, a strain-specific diagnosis method is required for efficient medication procedures.

### 2.4 Iron acquisition systems

The ExPEC strains contain a secondary metabolite such as siderophores promote bacterial colonization and biofilm formation. Under the iron-limited conditions, the *E. coli* strains showed reduced colonies and biovolume (biofilm formation). In ExPEC, the bacterial cells uptakes the extracellular iron from the blood and infected sites in the host. The iron acquisition systems in the ExPEC strains are classified into, enterobactin, aerobactin, salmochelin, and yersiniabactin. (i) The enterobactin encodes *entEBG* is studied with the chicken experimental model have shown a virulence mechanism such as immune evasion and extracellular iron uptake. (ii) the salmochelin (encodes *iroBCDEN*), the *iroB* genes alter the strong hydrophobic cell membrane to hydrophilic properties which promote extracellular iron uptakes (Sorsa et al., 2003). The *iroN* gene in the *E. coli* strain (UTI89) has shown an increased expression in the internal bacterial communities in the infection host epithelial tissues. (iii) the aerobactin (encoded by *iucABCD*) is more efficient than other uptake proteins, a very low amount of aerobactin expression in ExPEC strains uptakes sufficient quantities of extracellular iron from the transferrin and also interacts with albumin in host blood than other iron acquisition proteins (Reigstad et al., 2007). (iv) The excess uptake of iron turns to a toxic cellular effect, interestingly, the yersinabactin siderophores play a major role in the UTI which promotes biofilm formation in the host bladder and suppress the host immune mechanism by blocking the Haber-Weiss, these reactions promote reactive oxygen species (ROS) and produce hydroxyl radicals in the presence of excessive iron and causes DNA damage and lipid peroxidation in invading cells (Fasnacht and Polacek, 2021; Mello Filho and Meneghini, 1984). For instance, the Fur (Ferric uptake regulator) proteins regulate the iron homeostasis in cells. Once after attaining sufficient intracellular iron uptake, Fur coordinates the Fe^2+^to a specific DNA sequence called Fur box. This Fe^2+^to specific DNA-Fur box interaction further represses the genes of siderophore biosynthesis and its role in extracellular iron acquisition (Sandy and Butler, 2009). Overall, iron acquisition proteins (enterobactin, salmochelin, yesneriabactin, and aerobactin) possess iron uptake, bacterial colonization, and biofilm formation. Interestingly, the yesneriabactin siderophore showed an additional function in evading host immune defense and turn to promote pathogenicity (Chaturvedi et al., 2012).

### 2.5 ExPEC virulence toxins

ExPEC strains produce certain toxins such as α-hemolysin, autotransporter toxin, vacuolating autotransporter protein, and serin protease to disrupt the host immune defense mechanism and damage host cells (Nichols et al., 2016). The α-hemolysin toxins were secreted by the HlyA protein which is also known as type 1 secretion protein. It contains oligosaccharides on the N terminal which targets the β2 integrals on the host immune cells (Ristow and Welch, 2016). Also, hemolysin creates pores on the host cell membrane and promotes the arrival of cations and water which leads to the osmotic lysis of cells. To sum up, the complete functions of α-hemolysin toxins, toxin detection, and quantification are yet to be studied extensively. Similar to α-hemolysin toxins, epidemiological studies showed that cytotoxic necrotizing factor 1 (CNF1) was found in 48 % of the UTIs and showed higher epithelial-cell invasion in host tissue (Welch, 2017). Then, the rest toxins such as serine protease, secreted autotransporter toxins, and vacuolating auto-transporter proteins are grouped into Type V secretions. The secreted autotransporter toxins (sat) encode *sat* genes like α-hemolysin toxins and P pili. Previous studies on *sat* mutants have shown no significant differences in bacterial colonization and biofilm formation (Guyer et al., 2000). Interestingly, ExPECs toxin sat rearranged the F-actins in the epithelial cells, monolayer damage, departing host cell junctions, and exfoliation of urothelial cells in the UTI model (Toloza et al., 2015). The vacuolating autotransporter proteins and serin protease toxins were yet to be studied in the UTI model. Furthermore, the toxins are synthesized and tested for their chelating agents and inhibiting molecules to mitigate ExPEC infections.

## 3 Diagnostic mechanisms

### 3.1 Culture method

The bacterial culture method of identification has been used to detect intestinal and extraintestinal pathogenic bacteria in routine diagnostic laboratories (Najeeb et al., 2015). To date, standard quantitative urine culture is the “gold standard” test which is reliable with 85 % specificity and 95 % sensitivity (Abubakar et al., 2023; Perkins et al., 2012). This method can distinguish well the ExPEC from other extraintestinal pathogens (Abubakar et al., 2023). Colistin and nalidixic acid agar/MacConkey agar (CNA/MAC) are used to inoculate the urine samples and the morphology and colony forming unit (CFU) is possible to be recorded (Wojno et al., 2020). Particularly, MAC is most likely providing information to identify the pathogen or to isolate gram-negative ExPEC. Blood agar is also used to support the growth of aerobic ExPEC. This method involves the streaking of urine samples on the enrichment and selective medium. Even though this method has been exploited as a gold standard protocol for UTI detection, prolonged time is required to report the test result. At least 48 h is required to decide the result, which leads to delays in treatment and makes it unsuitable for prompt therapy of uncomplicated as well as leading to a considerable burden on economy patients (Liu et al., 2021; Schmiemann et al., 2010). Gram-stained microscopy can be used in parallel to the culture technique to increase diagnostic precision. However it's difficult to make a general statement by using Urine microscopy alone for UTI detection (Whiting et al., 2006). Immersion culture media that exploits plastic rods coated with CLED agar and MacConkey agar can be used detection of ExPEC (Winkens et al., 2003). However, the specificity and sensitivity value of this method are not reproducible from laboratory to laboratory. Studies exhibited that the sensitivity and specificity of this assay ranged between 66 and 80 % and 88–98 % respectively at a 95 % confidence interval (CI) (Schmiemann et al., 2010). If a negative nitrite test is found on the female patient, the sensitivity of the assay may be reduced to 55–74 %. On the other hand, this technique is not reliable for the detection of CFU <10^4^ per million litter (Gatermann et al., 2005). Additionally, the culture method requires special care during the collection, preservation, and transportation of urine samples to avoid the death and proliferation of bacteria and contamination (Bartges, 2004). Recent studies showed that the above-mentioned culture media are not sufficient to differentiate ExPEC from non-pathogenic *E. coli* and other uropathogens bacteria (Perry, 2017). To this end, various types of culture media have been investigated, for instance, sorbitol MacConkey agar (SMAC) can be used to detect ExPEC in strain levels such as enterohemorrhagic *E. coli* which can ferment sorbitol and produce β-d-glucuronidase (Spurbeck et al., 2012). Adding cefixime and tellurite to SMAC (CT-SMAC), potentially increase the stringency power of the medium (Koga et al., 2014). Other specific media such as Rainbow agar and CHROMagar® have been devised to differentiate Shiga toxin-producing/ Enterohaemorrhagic *E. coli* (STEC/EHEC) from ExPEC and Salmonella (Flament-Simon et al., 2020). Even though different modifications to the culture media have been made, it is labor intensive, inability to detect nonviable bacteria, the prolonged time required for definitive diagnosis, additional procedures needed for serotyping and antibiotic resistance information, and low detection limit, as well as the need for experienced technicians and well-equipped microbiology laboratory, impend to exploit it in routine medical laboratories (Hajar et al., 2011).

### 3.2 Molecular detection

Several molecular detections approach to improve the diagnosis of ExPEC, including Polymerase Chain Reaction (PCR) have been recommended. The molecular method allows for the differentiation of ExPEC beyond the subspecies level to determine the outbreak and transmission routes (Caméléna et al., 2019). Identifying the specific virulence gene as the molecular marker is the first step in designing molecular diagnostic assays. In the past year various marker genes were identified to detect pathogenic *E. coli*, for instance, α-*hly* (α *hemolysin*), LifA (lymphocyte inhibitory factor A), Efa1 (EHEC factor for adherence 1), EDL933 and SpLE3 genes in STEC strain, *ent, esc* (*encode for* enterohemolysin), *bfpB, stx*_2,_ *stx*_1_, estIb, *estIa, elt, invE, astA, aggR, pic, ent* were identified as maker genes to detect the Shiga toxin-producing *E. coli* (STEC), enteropathogenic *E. coli* (EPEC), enterotoxigenic *E. coli* (ETEC), enteroaggregative *E. coli* (EAEC), enteroinvasive *E. coli* (EIEC), and diffusely adherent *E. coli* (DAEC) (Ghanbarpour and Oswald, 2010; Nicolas-Chanoine et al., 2014; Van Belkum et al., 2001). These markers are mainly used to detect intestinal pathogenic *E. coli* at the strain level but identifying virulence markers for ExPEC has been undermined and very few markers have been identified so far. This hinders to devise of novel rapid and efficient molecular diagnostic assays for ExPEC. A few marker genes that encode toxins, siderophores, adhesins, invasives, and capsular antigens, connecting to extraintestinal infection are identified (Lindstedt et al., 2018). Additionally, the five crucial virulence factor (VF) encoding genes: afa/draBC, sfa/focDE, papAH and/or papC, iutA, and kpsM II, which have already been identified that used to distinguish ExPEC from other pathogens (Tóth et al., 2022). To confirm that the isolates are presumptive ExPEC, they should be positive or contain at least 2 of these marker genes. On the other hand, if the bacterial isolates are positive for at least three of the four VF-encoding genes (fyuA, yfcV, chuA, and vat), they are designated as uropathogenic *E. coli* (Mengistu and Mengesha, 2023; Seok et al., 2017; Xia et al., 2022). VF profiling is therefore beneficial to control the manifestation of ExPEC through performing efficient epidemiology research or confronting outbreaks, which can be achieved using molecular assays (Notomi et al., 2015). Even if the different genotyping methods of ExPEC have been applied, they have their advantages and drawbacks. Sequencing methods such as whole-genome sequencing (WGS) and Multi-Locus Sequence Typing (MLST) are advanced gold standard detection techniques with high power of discrimination but they are time-consuming and expensive which are not affordable in many developing worlds (Kogovšek et al., 2019; Kogovšek et al., 2019; Whiting et al., 2006; Zhao et al., 2010, 2013); and Pulsed-field gel electrophoresis (PFGE) is also recriminatory, but it needs a long time to provide results (Whiting et al., 2006). Whereas random PCR methods like conventional PCR and repetitive element palindromic PCR (REP-PC) are poorly reliable and lack discriminatory power (Seo et al., 2017; Shang et al., 2020). To overcome the shortcoming of conventional PCR, different molecular assays such as CRISPR-directed real-time PCR, multiplex PCR, droplet digital PCR (ddPCR) technology as well as commercial real-time PCR kits were devised so far by using the above-mentioned specific virulence factors to increase the sensitivity, specificity, and reliability of the assays (Shang et al., 2020). Through taking on the advantages of standard PCR which is easy, rapid, and inexpensive, and the high detection power of Multiple-Locus Variable (MLVA), combined MLVA with PCR method have developed to detect the typing ExPEC and intestinal pathogenic *E. coli* (Whiting et al., 2006). Notomi et al. (2015) also developed a multiplex PCR assay using VF genes for rapid profiling by ExPEC isolated from cattle. Another study devised Multiple-Locus Variable-Number Tandem-Repeat (VNTR) analysis using multiplex PCR and gel electrophoresis which is loftier in discrimination power than MLST and Diversi Lab REP-PCR to genotyping of *E. coli* particularly to distinguish pathogenic *E. coli* with its highly resemble bacteria (shigella spp.) as well as emergence of multidrug resistance (MDR) ExPEC (Shang et al., 2020). New cost-effective and efficient multiplex PCR was also devised to detect ST95 isolates that are non-susceptible to several antibiotics, like colistin and β-lactam drugs (Shang et al., 2020). The genes selected for incorporation into the MPCR, uidA, escV, bfpB, stx1, stx2, elt, estIa, estIb, invE, astA, aggR, and pic, were developed as well as Specific Novel multiplex ST1193-H64 PCR assay to ST1193-H64 that included primers for icd, fimH, and fumC was devised (Van Belkum et al., 2001). From a practical perspective, multiple studies decipher that rapid, efficient, and cost-effective identification drug resistant ExPEC is crucial for epidemiological and optimal tracking (Ravan et al., 2016; Song et al., 2005).

### 3.3 Loop-mediated isothermal amplification

**Fig. 2:**
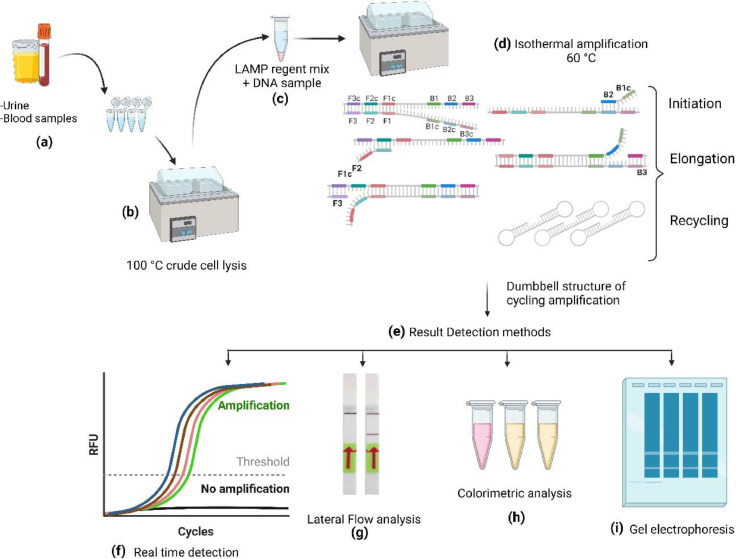
**Overview of the mechanism of LAMP assay for detecting ExPEC: (a)** The process begins with the collection of biological samples, **(b)** followed by crude cell lysis at 100 °C to release the DNA. **(c)** The released DNA is then mixed with a LAMP reagent and **(d)** subjected to DNA amplification, which includes steps such as initiation, elongation, and recycling of the DNA strands. The resulting dumbbell-shaped DNA structures allow continuous amplification, **(e)** The amplified DNA can be detected through various methods, **(f)** including real-time detection showing amplification curves, lateral flow analysis **(g)** lateral flow analysis, **(h)** colorimetric analysis: where a color change indicates a positive result, and **(i)** gel electrophoresis for separating and visualizing the amplified DNA fragments. Figure created using BioRender.com.

The advent of nucleic acid tests steps up the development of new affordable and point-care molecular diagnostic assays. As mentioned above, the combination of PCR with MLVA and MPCR has been devised to increase rapidity and efficiency, but they have still drawbacks in terms of cost and rapidity particularly, these techniques need highly equipped laboratories and trained personnel (Ramezani et al., 2018). Several studies evinced that isothermal amplification is a promising approach to overwhelm the cost and time of detection (Yinur et al., 2023; Hill et al., 2008). For instance, the most popular isothermal amplifications are strand displacement amplification (SDA) and self-sustained sequence replication (3SR) (Wang et al., 2012). Even though both methods use constant temperature to produce DNA copies from 5′ to 3′end, they still suffer from inadequacies and insufficiencies, they need sophisticated laboratory equipment. To advance this isothermal amplification, Notomi et al., successfully devised a novel method called loop-mediated isothermal amplification (LAMP). LAMP uses a set of four or six primers that bind the six or eight regions of the target segment of DNA consolidating its specificity (Wang et al., 2012). Since the development of LAMP, numerous studies on various infectious and non-infectious diseases have been carried out to confirm the result of amplification more simply and rapidly (Petrucci et al., 2021). To this end, recent studies exhibit that LAMP is significantly important to the diagnosis of pathogenic *E. coli* (Table 2). Hara-Kudo et al. developed three LAMP assays for specific, repeatable, and reproducible as well as rapid (results obtained in < 35 min) diagnosis of avian pathogenic *E. coli* by using three virulence genes; itA, traT, and ompT (Hara-Kudo et al., 2007). Some studies show that LAMP is prone to non-specific amplification and cross-contamination during reaction leading to false positive results (Petrucci et al., 2021). Integration of LAMP with microfluid and microchips can be alleviating this limitation and aggrandize the efficiency of the assay (Hara-Kudo et al., 2007). LAMP combined with microfluidic devices exhibited the potential of the assay for molecular diagnosis that can be ideal in terms of reducing costs, total time of analyses, and LAMP catastrophes (Hara-Kudo et al., 2007; Petrucci et al., 2021). LAMP-based microchip which includes full workflow DNA extraction, purification, amplification, and result detection would be an ideal molecular analysis platform for point care diagnosis assay of extra ExPEC infection (Fig. 2) (Table 2) (Etchebarne et al., 2017).

**Table 2:** LAMP assay development for detection of pathogenic *E. coli*:.

Pathogenic strains, isotypes	Virulence genes	Sample source	Result detection method	Assay time	Efficiency	Detection limit	Reference
APEC strains	APEC-associated virulence genes (sitA, ompT, traT) and an *E. coli*-specific gene (lamB)	animal swabs and tissues, and from environmental samples in commercial poultry farms	fluorescence was detected (FAM channel for the Roche LC480) during the cooling of the samples	<35min	85 %	10^3^ cells/mL	(Kogovšek et al., 2019)
*E. coli* O157 Strains	rfbE, stx1 and stx2	food samples	color change by the naked eye and by a fluorescence assay under UV	45 min	sensitivities of 100, 95.3, and 96.3 % for rfbE, stx1, and stx2 respectively, with specificity100 %	1 pg DNA	(Zhao et al., 2010)
*E. coli* O157	rfbE gene		SYBR Green I agarose gel	1 h	100 %	1.3 × 10^7^ CFU/ml	(Zhao et al., 2013)
Shigella and enteroinvasive *E. coli* (EIEC)	ipaH gene	stool samples	magnesium pyrophosphate turbidity	2 h	100 %	4 × 10^3^ CFU/ml	(Song et al., 2005)
*E. coli* O157:H7	Z3276 gene	Not directly stated	both turbidity and gel electrophoresis	1h	100 %	3 CFU/mL	(Ravan et al., 2016)
*E. coli*	16 s rRNA gene	urine samples	SYBR™	60 min	98 %	10^1^ CFU /mL	(Saengsawang et al., 2020)
*Eschericia coli* (*E. coli*)	*malB*	Urine specimens	SYBR green dye and agarose gel electrophoresis	1 hr	100 %	1.02 copies	(Ramezani et al., 2018)
urinary *E. coli*	*malB* gene	clinical samples, urine	Propidium iodide Sybr green	60 min	100 % sensitivity and 97 %specifivity	10 copies per reaction	(Hill et al., 2008)
Shiga toxin-producing *E. coli* (STEC)	Shiga toxin-producing E. coli (STEC)	*stx*_1_, *stx*_2_, and *eae*	LA-320C real-time turbidimeter with turbidity	1 h	100 %	1 to 20 CFU/reaction in pure culture and 10^3^ to 10^4^ CFU/g in spiked ground beef	(Wang et al., 2012)
*E. coli*	yaiO gen	Standard strain ATCC® 10,536™	Xylenol Orange (XO)	120 min	Not directly stated	1 CFU	(Jaroenram et al., 2019)
*E. coli* O157:H7	RNA of the rfbE gene	buffer, spinach, and ground beef samples.	lateral flow paper strip assay	2 h	Not directly stated	10 CFU/mL	(Petrucci et al., 2021)
Vero toxin (VT)-producing *E. coli*	VT1 and VT2 gene	food samples (beef and radish sprouts)	real-time turbidity at 650 nm direct observation of turbidity.	within 60 min	Not directly stated	0.7–2.2 cells per test	(Hara-Kudo et al., 2007)
infectious pathogens	pflB, LDH1, mecA, BckdE1, Fms14, Khe, CspA2, gehD, MstA, DiaA-2, ArcC, PMA1, VanC2, VanC1and TdcA	clinical blood, urine, wound, sputum, and stool samples.	Eriochrome Black T (EBT) dye	70–120 min	Sp 99.3 % Sn 65.5 %	Not directly stated	(Etchebarne et al., 2017)
Uropathies (*E. coli, Pseudomonas aeruginosa, Klebsiella pneumoniae, Proteus mirabilis*, and *Enterococcus faecalis*)	malB gene, oprL gene, rcsA gene, and Ef0027 gene,	urine samples	Calcein	Not directly stated	Sn 87.5 %- 100.0 % Sp 92.6 %- 96.6 %	between 104 and 105 CFU mL-	(Zeng et al., 2020)
*E. coli*	*E. coli* 23S ribosomal DNA (antimicrobial susceptibility testing marker)	Clinical urine	the digital real-time imaging instrument	30 min (directly from clinical samples)	Not directly stated	10^6^ copies/ml	(Schoepp et al., 2017)
*E. coli*	blaCTX-M-1 and blaCTX-M-15	Animal fecal sample	fluorescence detection	20 min	Not directly stated	8.5 and 9.8 copies per reaction	(Higgins et al., 2023)
*E. coli*	blaCTX-M9	Clinical Human isolates	SYBR Green	60 min	Not directly stated	10^−5^ng/mL	(Thirapanmethee et al., 2014)
Generic and Verocytotoxin-Producing *E. coli*	CDS encoding glycerate kinase	fecal samples	Loop-amp real-time turbidimeter	60min	Not directly stated	10^2^ CFU/mL	(Teh et al., ss 2014)

### 3.4 Immunological diagnostic assay for detection of ExPEC

The immune system of most people with ExPEC infections makes a lot of antibodies to fight this extra-intestinal pathogenic *E. coli* organism. However, this immune response does not work to remove the infection or keep it from returning after antibiotics destroy the bacterium. This immune response can be seen by serodiagnostic methods (Johnson et al., 2000).

**Fig. 3:**
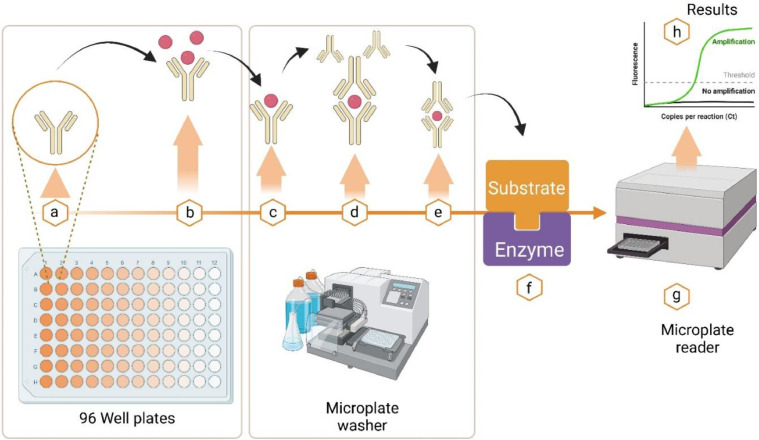
**ELISA test method for detection of ExPEC strains:** (a) Commercial ELISA test kits for ExPEC strains such as SafePath *E. coli* O:157 Microwell ELISA, REAGEN™ *E. coli* O157:H7 Elisa Test Kit, and 3M™ TECRA™ *E. coli* 0:157 were chosen to diagnose the ExPEC infection in humans, (b) the host test sample was loaded on the well plates which coated with a specific antibody (monoclonal or polyclonal) and allowed to incubate for few minutes and the excess samples were washed with wash buffer, and (c) the detection secondary antibody conjugated with enzyme is introduced to the same well and allowed for incubation, then the specific substrate was added and the plates were taken to fluorescent plate reader to identify the presence of ExPEC strains. Figure created using BioRender.com.

Some commercial immunodiagnostic assays like ELISA tests were developed and sold in the market for a year to detect ExPEC strains. However, there are some problems and limitations with the immunological detection of ExPEC, such as cross-reactivity, variability, and diversity of ExPEC strains and antigens. Therefore, it is very important to explore and study the virulence factor and antigenic proteins of ExPEC strains for developing an immunodiagnostic assay that is sensitive, accurate, and specific (Kunert Filho et al., 2015). The antigen preparation used affects the sensitivity and specificity of the test. Currently, ELISA-based serological detection of ExPEC strains uses several ExPEC strains antigenic proteins, including somatic antigen (on serogroup O), capsular antigen (on serogroups K), and flagella antigen type 1, fimbriae (on serogroup H) (Fratamico et al., 2016). Different study results indicate that there are 177 ‘O’ antigens, 100 ‘K’ antigens, 56 ‘H’ flagellar antigens, and 17 fimbriae antigens of ExPEC strains (Sora et al., 2021). Somatic ‘O’ antigens are largely part of endotoxin (lipopolysaccharide) in the outer bacterial membrane, and they account for most of the antigenic variability (Kunert Filho et al., 2015). A large portion of the ExPEC strains that belong to the ‘O’ group from different parts of the world are serogroup 01. 04.02. 03.06. 08.11. 15.36. 18.35. 50.21.64. 71.74. 75.78. 87.95. 100.88.103. 109.119.115. 132.141 and 152. The most immunogenic or antigenic serogroups among these are O1, O2, O8, O35, and O78 (Joensen et al., 2015). Another antigen that enables *E. coli* to adhere to the cell surface is a fimbrial antigen or pili antigen. Type 1 fimbriae, curli fimbriae, and a new fimbrial cluster of the Sfimbrial that attaches to avian tracheal cells; F1C, that attaches to buccal epithelial cells; Dr fimbriae, that remains in kidney tissue are found in most ExPEC. H antigens are the flagellar antigens on the surface of most ExPEC strains, and this antigen gives ExPEC motility (Rice et al., 2006). The capsular antigen (K-antigens) is composed of polysaccharides and is of polysaccharides and is mainly required in ExPEC strains to prevent the host organism's immune system, without this antigen is usually non-pathogenic. K-antigens presence in Gram-negative bacteria shows the main mechanism of evading the host phagocytosis (Willis and Whitfield, 2013). The most potential cases of neonatal meningitis, urinary tract infections, and septicemia are the ExPEC serotypes O1:K1, O2:K1, and O18:K1 (Moxley, 2022). Recently, five highly immunogenic virulence factors such as adhesins, invasins, iron uptake factors, and protectin, were expressed in ExPEC strains and used as antigenic proteins in the form of Oantigen, H antigen, and K antigen as well as Fimbiria antigen to find serologic immune responses against these virulence factors in the patient's sera (Sora et al., 2021). ExPEC can be detected by different immunological diagnosis methods, such as enzyme-linked immunosorbent assay (ELISA), immunofluorescence assay (IFA), immunochromatographic assay (ICA), or lateral flow assay (LFA). These methods use different types of labels (enzymes, fluorophores, colloidal gold) and different types of antibodies (monoclonal or polyclonal) to detect or estimate ExPEC antigens in a sample. Among these, Enzyme-linked immunosorbent assay (ELISA) ELISA is widely used as a sensitive and convenient diagnostic tool for finding specific pathogens' antigens or their specific antibodies. The principle of ELISA is the binding of an antigen of ExPEC to its specific antibody. The test is done on blood or urine and measures the number of specific antibodies specific to the ExPEC strain's antigenic proteins (Durant et al., 2007). For example, currently, an ELISA assay was developed to detect SinH which is an autotransporter protein involved in ExPEC colonization and virulence. The steps are as follows: (1)Coat the wells of a 96-well plate with anti-SinH monoclonal antibodies that attach to the SinH antigen, (2) add the sample (e.g., serum or urine) that may have the SinH antigen and incubate, (3) remove unbound components and add anti-SinH polyclonal antibodies that bind to a different epitope of the SinH antigen and are conjugated to horseradish peroxidase (HRP) (4) add tetramethylbenzidine (TMB) substrate that reacts with HRP to produce color (5) use a plate reader at 450 nm wavelength to measure the color amount (OD) of each well and compare it with a standard curve made with known quantities of recombinant SinH protein, (6) determine the quantity or concentration of the SinH antigen in the sample using the standard curve equation (Xing et al., 2023). Generally, the ELISA-based ExPEC strain detection principle is shown in Fig. 1 (Durant et al., 2007). Presently, there are many types of ELISA formats, such as indirect, direct, sandwich, competitive, and a new type, multiple and portable ELISA. ExPEC strains can be identified by many commercial ELISA kits, such as *E. coli* F5 ELISA, SafePath *E. coli* O:157 Microwell ELISA, TECRA *E. coli* 0:157, REAGEN *E. coli* O157:H7 Elisa Kit. For example, the BIO K 345 *E. coli* F5 ELISA Kit measures the antibodies of the F5 (K99) pilus antigen of ExPEC strains, and also *E. coli*- P ELISA Kit is a sandwich enzyme immunoassay technique that detects the P fimbrial adhesins of ExPEC strain serotype F11P antigen in serum, plasma, and tissue homogenates (Kunert Filho et al., 2015). According to Pitout (2012), an indirect haemagglutination test, a common method for identifying antibodies against ExPEC strains antigenic proteins such as fimbrial antigen, capsule antigen, and somatic antigens, is less sensitive than the ELISA test (Fig. 3).

## 4 Conclusion and future perspectives

Taking into account, the molecular mechanism of ExPEC pathogenesis in the UTI is well studied, the virulence pathogens and their virulence were factors, and virulence genes were used to develop the various strain-specific diagnoses in UTI. Diagnosis such as cultures-based assay, molecular virulence gene identification, loop-mediated isothermal amplification techniques, and ELISA were clinically practiced in identifying the ExPEC strains in UTI. In addition, *E. coli* has certain characteristics such as the expression of *fim* or *pap*, but both genes never express at the same time, these features added advantages in treating infections. However, the ExPEC pathogens undergo asymptomatic colonization in the intestine which remains a major research gap in diagnosis. Being asymptomatic and the absence of virulence genes makes the pathogens escape from notice and cause infections in humans. Researchers developed the closely related phylogeny to detect the pathogens in food origins and to knock out the ExPEC from the food cycle. ExPEC/UPEC gained more importance in poultry meat and poultry products around the world.

## CRediT author contribution statement

**Deenadayalan Karaiyagowder Govindarajan:** Conceptualization; Data curation; Methodology; Resources; Software; Validation; Visualization; Roles/Writing - original draft; Writing -review & editing. **Binaim Moges Eskeziyaw**: Conceptualization; Data curation; Methodology; Resources; Software; Validation; Visualization; Roles/Writing - original draft; Writing -review & editing. **Kumaravel Kandaswamy:** Conceptualization; Formal analysis; Funding acquisition; Investigation; Project administration; Resources; Software; Supervision; Validation; Visualization; Roles/Writing - original draft; Writing - review & editing. **Degisew Yinur Mengistu:** Conceptualization; Formal analysis; Investigation; Project administration; Resources; Software; Supervision; Validation; Visualization; Roles/Writing - original draft; Writing - review & editing.

## Declaration of competing interest

The authors declare that they have no known competing financial interests or personal relationships that could have appeared to influence the work reported in this paper.

## Data Availability

Data will be made available on request.

## References

[bib0001] Abreu A.G., Barbosa A.S. (2017). How Escherichia coli circumvent complement-mediated killing. Front. Immunol..

[bib0002] Abubakar N., Aliyu M., Jibril M., Mohammed Y. (2023). Polymerase chain reaction detection of haemolysin D gene (hlyD) in uropathogenic Escherichia coli as a novel diagnostic test for urinary tract infection. Afric. J. Clin. Exp. Microbiol..

[bib0003] Balsalobre C., Morschhäuser J., Jass J., Hacker J.r., Uhlin B.E. (2003). Transcriptional analysis of the sfa determinant revealing multiple mRNA processing events in the biogenesis of S fimbriae in pathogenic Escherichia coli. J. Bacteriol..

[bib0004] Bartges J.W. (2004). Diagnosis of urinary tract infections. Vet. Clin.: Small Anim. Practice.

[bib0005] Brede C.M., Shoskes D.A. (2011). The etiology and management of acute prostatitis. Nature Rev. Urol..

[bib0006] Caméléna F., Birgy A., Smail Y., Courroux C., Mariani-Kurkdjian P., Le Hello S., Bidet P. (2019). Rapid and simple universal Escherichia coli genotyping method based on multiple-locus variable-number tandem-repeat analysis using single-tube multiplex PCR and standard gel electrophoresis. Appl. Environ. Microbiol..

[bib0007] Chaturvedi K.S., Hung C.S., Crowley J.R., Stapleton A.E., Henderson J.P. (2012). The siderophore yersiniabactin binds copper to protect pathogens during infection. Nat. Chem. Biol..

[bib0008] Clermont O., Bonacorsi S., Bingen E. (2000). Rapid and simple determination of the Escherichia coli phylogenetic group. Appl. Environ. Microbiol..

[bib0009] Dehghani B., Mottamedifar M., Khoshkharam-Roodmajani H., Hassanzadeh A., Zomorrodian K., Rahimi A. (2016). SDS-PAGE analysis of the outer membrane proteins of uropathogenic Escherichia coli isolated from patients in different wards of Nemazee Hospital, Shiraz, Iran. Iran. J. Med. Sci..

[bib0010] Dobrut A., Ochońska D., Brzozowska E., Górska S., Kaszuba-Zwoinska J., Gołda-Cępa M., Brzychczy-Wloch M. (2022). Molecular characteristic, antibiotic resistance, and detection of highly immunoreactive proteins of Group B Streptococcus strains isolated from urinary tract infections in polish adults. Front. Microbiol..

[bib113] Durant L., Metais A., Soulama-Mouze C., Genevard J.M., Nassif X., Escaich S. (2007). Identification of candidates for a subunit vaccine against extraintestinal pathogenic Escherichia coli. Infect. Immun..

[bib0011] Erman A., Lakota K., Mrak-Poljsak K., Blango M.G., Krizan-Hergouth V., Mulvey M.A., Veranic P. (2012). Uropathogenic Escherichia coli induces serum amyloid a in mice following urinary tract and systemic inoculation. PLoS. One.

[bib0012] Etchebarne B.E., Li Z., Stedtfeld R.D., Nicholas M.C., Williams M.R., Johnson T.A., Tiedje J.M. (2017). Evaluation of nucleic acid isothermal amplification methods for human clinical microbial infection detection. Front. Microbiol..

[bib0013] Eto D.S., Jones T.A., Sundsbak J.L., Mulvey M.A. (2007). Integrin-mediated host cell invasion by type 1–piliated uropathogenic Escherichia coli. PLoS Pathog..

[bib0014] Fasnacht M., Polacek N. (2021). Oxidative stress in bacteria and the central dogma of molecular biology. Front. Mol. Biosci..

[bib0015] Flament-Simon S.-C., De Toro M., García V., Blanco J.E., Blanco M., Alonso M.P., Blanco J. (2020). Molecular characteristics of extraintestinal pathogenic E. coli (ExPEC), uropathogenic E. coli (UPEC), and multidrug resistant E. coli isolated from healthy dogs in Spain. Whole genome sequencing of canine ST372 isolates and comparison with human isolates causing extraintestinal infections. Microorganisms..

[bib0016] Foxman B. (2010). The epidemiology of urinary tract infection. Nature Rev. Urol..

[bib0017] Foxman B., Brown P. (2003). Epidemiology of urinary tract infections: transmission and risk factors, incidence, and costs. Infectious Disease Clinics.

[bib0018] François M., Hanslik T., Dervaux B., Le Strat Y., Souty C., Vaux S., Heym B. (2016). The economic burden of urinary tract infections in women visiting general practices in France: a cross-sectional survey. BMC. Health Serv. Res..

[bib0019] Frank C., Werber D., Cramer J.P., Askar M., Faber M., an der Heiden M., Spode A. (2011). Epidemic profile of Shiga-toxin–producing Escherichia coli O104: H4 outbreak in Germany. N. England J. Med..

[bib107] Fratamico P.M., DebRoy C., Needleman D.S. (2016). Emerging approaches for typing, detection, characterization, and traceback of Escherichia coli. Fron. Microbiol..

[bib0020] Gatermann S., Fünfstück R., Handrick W., Podbielski A. (2005).

[bib0021] Gerdes K., Christensen S.K., Løbner-Olesen A. (2005). Prokaryotic toxin–antitoxin stress response loci. Nature Rev. Microbiol..

[bib0022] Ghanbarpour R., Oswald E. (2010). Phylogenetic distribution of virulence genes in Escherichia coli isolated from bovine mastitis in Iran. Res. Vet. Sci..

[bib0023] Govindarajan D.K., Kandaswamy K. (2022). Virulence factors of uropathogens and their role in host pathogen interactions. Cell Surf..

[bib0024] Govindarajan D.K., Kandaswamy K. (2023). Antimicrobial peptides: a small molecule for sustainable healthcare applications. Med. Microecol..

[bib0025] Govindarajan D.K., Meghanathan Y., Sivaramakrishnan M., Kothandan R., Muthusamy A., Seviour T.W., Kandaswamy K. (2022). Enterococcus faecalis thrives in dual-species biofilm models under iron-rich conditions. Arch. Microbiol..

[bib0026] Govindarajan D.K., Viswalingam N., Meganathan Y., Kandaswamy K. (2020). Adherence patterns of Escherichia coli in the intestine and its role in pathogenesis. Med. Microecol..

[bib0027] Guyer D.M., Henderson I.R., Nataro J.P., Mobley H.L. (2000). Identification of sat, an autotransporter toxin produced by uropathogenic Escherichia coli. Mol. Microbiol..

[bib0028] Hacker J., Kestler H., Hoschützky H., Jann K., Lottspeich F., Korhonen T. (1993). Cloning and characterization of the S fimbrial adhesin II complex of an Escherichia coli O18: K1 meningitis isolate. Infect. Immun..

[bib0029] Hajar F., Taleb M., Aoun B., Shatila A. (2011). Dipstick urine analysis screening among asymptomatic school children. N. Am. J. Med. Sci..

[bib0030] Hara-Kudo Y., Nemoto J., Ohtsuka K., Segawa Y., Takatori K., Kojima T., Ikedo M. (2007). Sensitive and rapid detection of Vero toxin-producing Escherichia coli using loop-mediated isothermal amplification. J. Med. Microbiol..

[bib0031] Hill J., Beriwal S., Chandra I., Paul V.K., Kapil A., Singh T., Jahnukainen T. (2008). Loop-mediated isothermal amplification assay for rapid detection of common strains of Escherichia coli. J. Clin. Microbiol..

[bib0032] Jaroenram W., Cecere P., Pompa P.P. (2019). Xylenol orange-based loop-mediated DNA isothermal amplification for sensitive naked-eye detection of Escherichia coli. J. Microbiol. Methods.

[bib108] Joensen K.G., Tetzschner A.M., Iguchi A., Aarestrup F.M., Scheutz F. (2015). Rapid and easy in silico serotyping of Escherichia coli isolates by use of whole-genome sequencing data. J. Clin. Microbiol..

[bib0033] Johnson J.R., Stell A.L., Delavari P. (2001). Canine feces as a reservoir of extraintestinal pathogenic Escherichia coli. Infect. Immun..

[bib105] Johnson J.R., Stell A.L., Scheutz F., O’Bryan T.T., Russo T.A., Carlino U.B., Gaastra W. (2000). Analysis of the F antigen-specific papA alleles of extraintestinal pathogenic Escherichia coli using a novel multiplex PCR-based assay. Infect. Immun.

[bib0034] Justice S.S., Hunstad D.A., Seed P.C., Hultgren S.J. (2006). Filamentation by Escherichia coli subverts innate defenses during urinary tract infection. Proc. Natl. Acad. Sci..

[bib0035] Kadirvelu L., Sivaramalingam S.S., Jothivel D., Chithiraiselvan D.D., Govindarajan D.K., Kandaswamy K. (2024). A review on antimicrobial strategies in mitigating biofilm-associated infections on medical implants. Curr. Res. Microb. Sci..

[bib0036] Keogh D., Lam L.N., Doyle L.E., Matysik A., Pavagadhi S., Umashankar S., Ng S.P. (2018). Extracellular electron transfer powers Enterococcus faecalis biofilm metabolism. mBio.

[bib0037] Khalid S., Bond P.J., Carpenter T., Sansom M.S. (2008). OmpA: gating and dynamics via molecular dynamics simulations. Biochimica et Biophysica Acta (BBA)-Biomembranes.

[bib0038] Koga V.L., Tomazetto G., Cyoia P.S., Neves M.S., Vidotto M.C., Nakazato G., Kobayashi R.K. (2014). Molecular screening of virulence genes in extraintestinal pathogenic Escherichia coli isolated from human blood culture in Brazil. Biomed. Res. Int..

[bib0039] Kogovšek P., Ambrožič-Avguštin J., Dovč A., Dreo T., Hristov H., Krapež U., Žel J. (2019). Loop-mediated isothermal amplification: rapid molecular detection of virulence genes associated with avian pathogenic Escherichia coliin poultry. Poult. Sci..

[bib0040] Korhonen T.K., Parkkinen J., Hacker J., Finne J., Pere A., Rhen M., Holthöfer H. (1986). Binding of Escherichia coli S fimbriae to human kidney epithelium. Infect. Immun..

[bib0041] Krishnan S., Prasadarao N.V. (2012). Outer membrane protein A and OprF: versatile roles in Gram-negative bacterial infections. FEBS J..

[bib106] Kunert Filho H.C., Carvalho D., Grassotti T.T., Soares B.D., Rossato J.M., Cunha A.C., Brito B.G. (2015). Avian pathogenic Escherichia coli-methods for improved diagnosis. J. World’s Poult. Sci..

[bib0042] Lindstedt B.-A., Finton M.D., Porcellato D., Brandal L.T. (2018). High frequency of hybrid Escherichia coli strains with combined Intestinal Pathogenic Escherichia coli (IPEC) and Extraintestinal Pathogenic Escherichia coli (ExPEC) virulence factors isolated from human faecal samples. BMC Infect. Dis..

[bib0043] Liu H., Li Z., Shen R., Li Z., Yang Y., Yuan Q. (2021). Point-of-care pathogen testing using photonic crystals and machine vision for diagnosis of urinary tract infections. Nano Lett..

[bib0044] Luna-Pineda V.M., Moreno-Fierros L., Cázares-Domínguez V., Ilhuicatzi-Alvarado D., Ochoa S.A., Cruz-Córdova A., Xicohtencatl-Cortes J. (2019). Curli of uropathogenic Escherichia coli enhance urinary tract colonization as a fitness factor. Front. Microbiol..

[bib0045] Mann R., Mediati D.G., Duggin I.G., Harry E.J., Bottomley A.L. (2017). Metabolic adaptations of uropathogenic E. coli in the urinary tract. Front. Cell Infect. Microbiol..

[bib0046] Meganathan Y., Govindarajan D.K., Sivaramakrishnan M., Kumaravel K. (2023). Sustainable Digital Technologies for Smart Cities.

[bib0047] Mello Filho A.C., Meneghini R. (1984). In vivo formation of single-strand breaks in DNA by hydrogen peroxide is mediated by the Haber-Weiss reaction. Biochimica et Biophysica Acta (BBA)-Gene Struct. Expression.

[bib0048] Mengistu D.Y., Mengesha Y. (2023). New approaches for severity intervention and rapid diagnosis of enterohemorrhagic Escherichia coli: a review. All. Life.

[bib0049] Mike L.A., Smith S.N., Sumner C.A., Eaton K.A., Mobley H.L. (2016). Siderophore vaccine conjugates protect against uropathogenic Escherichia coli urinary tract infection. Proc. Natl. Acad. Sci..

[bib0050] Morschhäuser J., Hoschützky H., Jann K., Hacker J. (1990). Functional analysis of the sialic acid-binding adhesin SfaS of pathogenic Escherichia coli by site-specific mutagenesis. Infect. Immun..

[bib109] Moxley R.A. (2022). Enterobacteriaceae: Escherichia. Vet. Microbiol..

[bib0051] Najeeb S., Munir T., Rehman S., Hafiz A., Gilani M., Latif M. (2015). Comparison of urine dipstick test with conventional urine culture in diagnosis of urinary tract infection. J. Coll. Physicians Surg. Pak..

[bib0052] Nhu N.T.K., Phan M.-D., Forde B.M., Murthy A.M., Peters K.M., Day C.J., Jennings M.P. (2019). Complex multilevel control of hemolysin production by uropathogenic Escherichia coli. mBio.

[bib0053] Nichols K.B., Totsika M., Moriel D.G., Lo A.W., Yang J., Wurpel D.J., Ulett G.C. (2016). Molecular characterization of the vacuolating autotransporter toxin in uropathogenic Escherichia coli. J. Bacteriol..

[bib0054] Nicolas-Chanoine M.-H., Bertrand X., Madec J.-Y. (2014). Escherichia coli ST131, an intriguing clonal group. Clin. Microbiol. Rev..

[bib0055] Norton, J.P., & Mulvey, M.A. (2012). Toxin-antitoxin systems are important for niche-specific colonization and stress resistance of uropathogenic Escherichia coli. 10.1371/journal.ppat.1002954 PMC3464220 23055930

[bib0056] Notomi T., Mori Y., Tomita N., Kanda H. (2015). Loop-mediated isothermal amplification (LAMP): principle, features, and future prospects. J. Microbiol..

[bib0057] Ochman H., Selander R.K. (1984). Standard reference strains of Escherichia coli from natural populations. J. Bacteriol..

[bib104] Parkkinen J., Finne J., Achtman M., Väisänen V., Korhonen T.K. (1983). Escherichia coli strains binding neuraminyl α2–3 galactosides. Biochem. Biophys. Res. Commun..

[bib0058] Perkins J., Perkins K., Vilke G.M., Almazroua F.Y. (2012). Is culture-positive urinary tract infection in febrile children accurately identified by urine dipstick or microanalysis?. J. Emerg. Med..

[bib0059] Perry J.D. (2017). A decade of development of chromogenic culture media for clinical microbiology in an era of molecular diagnostics. Clin. Microbiol. Rev..

[bib0060] Petrucci S., Costa C., Broyles D., Kaur A., Dikici E., Daunert S., Deo S.K. (2021). Monitoring pathogenic viable E. coli O157: H7 in food matrices based on the detection of RNA using isothermal amplification and a paper-based platform. Anal. Chem..

[bib111] Ragupathi H., Pushparaj M.M., Gopi S.M., Govindarajan D.K., Kandaswamy K. (2024). Biofilm matrix: a multifaceted layer of biomolecules and a defensive barrier against antimicrobials. Archives of Microbiology.

[bib0061] Rahdar M., Rashki A., Miri H.R., Ghalehnoo M.R. (2015). Detection of pap, sfa, afa, foc, and fim adhesin-encoding operons in uropathogenic Escherichia coli isolates collected from patients with urinary tract infection. Jundishapur. J. Microbiol..

[bib0062] Ramezani R., Parizi Z.K., Ghorbanmehr N., Mirshafiee H. (2018). Rapid and simple detection of Escherichia coli by loop-mediated isothermal amplification assay in urine specimens. Avicenna J. Med. Biotechnol..

[bib0063] Ravan H., Amandadi M., Sanadgol N. (2016). A highly specific and sensitive loop-mediated isothermal amplification method for the detection of Escherichia coli O157: H7. Microb. Pathog..

[bib0064] Reigstad C.S., Hultgren S.J., Gordon J.I. (2007). Functional genomic studies of uropathogenic Escherichia coli and host urothelial cells when intracellular bacterial communities are assembled. J. Biol. Chem..

[bib112] Rice J.C., Peng T., Kuo Y.F., Pendyala S., Simmons L., Boughton J., Nowicki B.J. (2006). Renal allograft injury is associated with urinary tract infection caused by Escherichia coli bearing adherence factors. Am. J. Transplant..

[bib0065] Ristow L.C., Welch R.A. (2016). Hemolysin of uropathogenic Escherichia coli: a cloak or a dagger?. Biochimica et Biophysica Acta (BBA)-Biomembranes.

[bib0066] Russo T.A., Johnson J.R. (2000). Proposal for a new inclusive designation for extraintestinal pathogenic isolates of Escherichia coli: exPEC. J. Infect. Dis..

[bib0067] Saengsawang N., Ruang-Areerate T., Kesakomol P., Thita T., Mungthin M., Dungchai W. (2020). Development of a fluorescent distance-based paper device using loop-mediated isothermal amplification to detect Escherichia coli in urine. Analyst.

[bib0068] Sandy M., Butler A. (2009). Microbial iron acquisition: marine and terrestrial siderophores. Chem. Rev..

[bib0069] Sarowska J., Futoma-Koloch B., Jama-Kmiecik A., Frej-Madrzak M., Ksiazczyk M., Bugla-Ploskonska G., Choroszy-Krol I. (2019). Virulence factors, prevalence and potential transmission of extraintestinal pathogenic Escherichia coli isolated from different sources: recent reports. Gut. Pathog..

[bib0070] Schmiemann G., Kniehl E., Gebhardt K., Matejczyk M.M., Hummers-Pradier E. (2010). The diagnosis of urinary tract infection: a systematic review. Deutsches Ärzteblatt Int..

[bib0071] Schoepp N.G., Schlappi T.S., Curtis M.S., Butkovich S.S., Miller S., Humphries R.M., Ismagilov R.F. (2017). Rapid pathogen-specific phenotypic antibiotic susceptibility testing using digital LAMP quantification in clinical samples. Sci. Transl. Med..

[bib0072] Seo J.H., Park B.H., Oh S.J., Choi G., Lee E.Y., Seo T.S. (2017). Development of a high-throughput centrifugal loop-mediated isothermal amplification microdevice for multiplex foodborne pathogenic bacteria detection. Sensor. Actuators B: Chem..

[bib0073] Seok Y., Joung H.-A., Byun J.-Y., Jeon H.-S., Shin S.J., Kim S., Kim M.-G. (2017). A paper-based device for performing loop-mediated isothermal amplification with real-time simultaneous detection of multiple DNA targets. Theranostics..

[bib0074] Servin A.L. (2014). Pathogenesis of human diffusely adhering Escherichia coli expressing Afa/Dr adhesins (Afa/Dr DAEC): current insights and future challenges. Clin. Microbiol. Rev..

[bib0075] Shang Y., Sun J., Ye Y., Zhang J., Zhang Y., Sun X. (2020). Loop-mediated isothermal amplification-based microfluidic chip for pathogen detection. Crit. Rev. Food Sci. Nutr..

[bib0076] Shanmugasundarasamy T., Govindarajan D.K., Kandaswamy K. (2022). A review on pilus assembly mechanisms in Gram-positive and Gram-negative bacteria. The Cell Surface.

[bib0077] Sivaramalingam S.S., Jothivel D., Govindarajan D.K., Kadirvelu L., Sivaramakrishnan M., Chithiraiselvan D.D., Kandaswamy K. (2024). Structural and functional insights of sortases and their interactions with antivirulence compounds. Curr. Res. Struct. Biol..

[bib0078] Song T., Toma C., Nakasone N., Iwanaga M. (2005). Sensitive and rapid detection of Shigella and enteroinvasive Escherichia coli by a loop-mediated isothermal amplification method. FEMS Microbiol. Lett..

[bib0079] Sora V.M., Meroni G., Martino P.A., Soggiu A., Bonizzi L., Zecconi A. (2021). Extraintestinal pathogenic Escherichia coli: virulence factors and antibiotic resistance. Pathogens..

[bib0080] Sorsa L.J., Dufke S., Heesemann J.r., Schubert S.r. (2003). Characterization of an iroBCDEN gene cluster on a transmissible plasmid of uropathogenic Escherichia coli: evidence for horizontal transfer of a chromosomal virulence factor. Infect. Immun..

[bib0081] Spencer J.D., Jackson A.R., Li B., Ching C.B., Vonau M., Easterling R.S., Becknell B. (2015). Expression and significance of the HIP/PAP and RegIIIγ antimicrobial peptides during mammalian urinary tract infection. PLoS. One.

[bib0082] Spurbeck R.R., Dinh P.C., Walk S.T., Stapleton A.E., Hooton T.M., Nolan L.K., Mobley H.L. (2012). Escherichia coli isolates that carry vat, fyuA, chuA, and yfcV efficiently colonize the urinary tract. Infect. Immun..

[bib0083] Stork C., Kovács B., Rózsai B., Putze J., Kiel M., Dorn Á., Kovács T. (2018). Characterization of asymptomatic bacteriuria Escherichia coli isolates in search of alternative strains for efficient bacterial interference against uropathogens. Front. Microbiol..

[bib0084] Subashchandrabose S., Mobley H.L. (2017). Virulence and fitness determinants of uropathogenic Escherichia coli. Urinary Tract Infect.: Mol. Pathogenesis Clin. Manag..

[bib0085] Sun Y., Wang X., Li J., Xue F., Tang F., Dai J. (2022). Extraintestinal pathogenic Escherichia coli utilizes the surface-expressed elongation factor Tu to bind and acquire iron from holo-transferrin. Virulence.

[bib0086] Teh C.S.J., Chua K.H., Lim Y.A.L., Lee S.C., Thong K.L. (2014). Loop-mediated isothermal amplification assay for detection of generic and verocytotoxin-producing Escherichia coli among indigenous individuals in Malaysia. Sci. World J..

[bib0087] Teng C.-H., Xie Y., Shin S., Di Cello F., Paul-Satyaseela M., Cai M., Kim K.S. (2006). Effects of ompA deletion on expression of type 1 fimbriae in Escherichia coli K1 strain RS218 and on the association of E. coli with human brain microvascular endothelial cells. Infect. Immun..

[bib0088] Thirapanmethee K., Pothisamutyothin K., Nathisuwan S., Chomnawang M.T., Wiwat C. (2014). Loop-mediated isothermal amplification assay targeting the blaCTX-M9 gene for detection of extended spectrum β-lactamase-producing Escherichia coli and Klebsiella pneumoniae. Microbiol. Immunol..

[bib0089] Toloza L., Giménez R., Fábrega M.J., Alvarez C.S., Aguilera L., Cañas M.A., Baldomà L. (2015). The secreted autotransporter toxin (Sat) does not act as a virulence factor in the probiotic Escherichia coli strain Nissle 1917. BMC Microbiol..

[bib0090] Tóth I., Bagyinszky E., Sváb D. (2022). Multiplex polymerase chain reaction typing scheme based on Escherichia coli O157: H7 Sakai prophage (Sp)-associated genes. Int. J. Infect. Dis..

[bib0091] Van Belkum A., Struelens M., de Visser A., Verbrugh H., Tibayrenc M. (2001). Role of genomic typing in taxonomy, evolutionary genetics, and microbial epidemiology. Clin. Microbiol. Rev..

[bib0092] Wang F., Jiang L., Ge B. (2012). Loop-mediated isothermal amplification assays for detecting Shiga toxin-producing Escherichia coli in ground beef and human stools. J. Clin. Microbiol..

[bib0093] Welch R.A. (2017). Uropathogenic Escherichia coli-associated exotoxins. Urinary Tract Infections: Mol. Pathogenesis Clin. Manag..

[bib0094] Whiting P., Westwood M., Bojke L., Palmer S., Richardson G., Cooper J., Kleijnen J. (2006). Clinical effectiveness and cost-effectiveness of tests for the diagnosis and investigation of urinary tract infection in children: a systematic review and economic model. Health Technol. Assess..

[bib0095] Willis L.M., Whitfield C. (2013). Structure, biosynthesis, and function of bacterial capsular polysaccharides synthesized by ABC transporter-dependent pathways. Carbohydr. Res..

[bib0096] Winkens R., Nelissen-Arets H., Stobberingh E. (2003). Validity of the urine dipslide under daily practice conditions. Fam. Pract..

[bib0097] Wojno K.J., Baunoch D., Luke N., Opel M., Korman H., Kelly C., Hindu S. (2020). Multiplex PCR based urinary tract infection (UTI) analysis compared to traditional urine culture in identifying significant pathogens in symptomatic patients. Urology..

[bib0098] Xia F., Cheng J., Jiang M., Wang Z., Wen Z., Wang M., Zhuge X. (2022). Genomics analysis to identify multiple genetic determinants that drive the global transmission of the pandemic ST95 lineage of Extraintestinal pathogenic escherichia coli (ExPEC). Pathogens..

[bib110] Xing G., Shang Y., Ai J., Lin H., Wu Z., Zhang Q., Lin L. (2023). Nanozyme-mediated catalytic signal amplification for microfluidic biosensing of foodborne bacteria. Anal. Chem..

[bib0099] Yinur D., Moges B., Hassen A., Tessema T.S. (2023). Loop mediated isothermal amplification as a molecular diagnostic assay: application and evaluation for detection of Enterohaemorrhagic Escherichia coli (O157: H7). Pract. Lab. Med..

[bib0100] Yu X., Gan Z., Wang Z., Tang X., Guo B., Du H. (2019). Increased iron availability aggravates the infection of Escherichia coli O157: H7 in mice. Biol. Trace Elem. Res..

[bib0101] Zeng Y., Liu M., Xia Y., Jiang X. (2020). Uracil-DNA-glycosylase-assisted loop-mediated isothermal amplification for detection of bacteria from urine samples with reduced contamination. Analyst.

[bib0102] Zhao X., Li Y., Wang L., You L., Xu Z., Li L., Yang L. (2010). Development and application of a loop-mediated isothermal amplification method on rapid detection Escherichia coli O157 strains from food samples. Mol. Biol. Rep..

[bib0103] Zhao X., Wang J., Forghani F., Park J.-H., Park M.-S., Seo K.-H., Oh D.-H. (2013). Rapid detection of viable Escherichia coli O157 by coupling propidium monoazide with loop-mediated isothermal amplification. J. Microbiol. Biotechnol..

